# Combinatorial Effects of Fatty Acid Elongase Enzymes on Nervonic Acid Production in *Camelina sativa*

**DOI:** 10.1371/journal.pone.0131755

**Published:** 2015-06-29

**Authors:** Dongxin Huai, Yuanyuan Zhang, Chunyu Zhang, Edgar B. Cahoon, Yongming Zhou

**Affiliations:** 1 National Key Laboratory of Crop Genetic Improvement and College of Plant Science and Technology, Huazhong Agricultural University, Wuhan, China; 2 Center for Plant Science Innovation and Department of Biochemistry, University of Nebraska-Lincoln, Lincoln, NE, United States of America; INRA, FRANCE

## Abstract

Very long chain fatty acids (VLCFAs) with chain lengths of 20 carbons and longer provide feedstocks for various applications; therefore, improvement of VLCFA contents in seeds has become an important goal for oilseed enhancement. VLCFA biosynthesis is controlled by a multi-enzyme protein complex referred to as fatty acid elongase, which is composed of *β*-ketoacyl-CoA synthase (KCS), *β*-ketoacyl-CoA reductase (KCR), *β*-hydroxyacyl-CoA dehydratase (HCD) and enoyl reductase (ECR). KCS has been identified as the rate-limiting enzyme, but little is known about the involvement of other three enzymes in VLCFA production. Here, the combinatorial effects of fatty acid elongase enzymes on VLCFA production were assessed by evaluating the changes in nervonic acid content. A *KCS* gene from *Lunaria annua* (*LaKCS*) and the other three elongase genes from *Arabidopsis thaliana* were used for the assessment. Five seed-specific expressing constructs, including *LaKCS* alone, *LaKCS* with *AtKCR*, *LaKCS* with *AtHCD*, *LaKCS* with *AtECR*, and *LaKCS* with *AtKCR* and *AtHCD*, were transformed into *Camelina sativa*. The nervonic acid content in seed oil increased from null in wild type camelina to 6-12% in *LaKCS*-expressing lines. However, compared with that from the *LaKCS*-expressing lines, nervonic acid content in mature seeds from the co-expressing lines with one or two extra elongase genes did not show further increases. Nervonic acid content from *LaKCS*, *AtKCR* and *AtHCD* co-expressing line was significantly higher than that in *LaKCS*-expressing line during early seed development stage, while the ultimate nervonic acid content was not significantly altered. The results from this study thus provide useful information for future engineering of oilseed crops for higher VLCFA production.

## Introduction

Very long chain fatty acids (VLCFAs) with chain lengths of 20 or more carbons are often modified, derivatized, or linked with other compounds to produce biologically active products that are involved in the biosynthesis of cuticular waxes, seed triacylglycerols and sphingolipids in plants [1]. VLCFAs, such as erucic acid (C22:1Δ13) and nervonic acid (C24:1Δ15), are an important renewable feedstock in plastic, cosmetic, nylon and lubricant industries [2–5]. Nervonic acid is also applied to the treatment of neurological diseases associated with multiple sclerosis, adrenoleukodystrophy and Zellweger syndrome [6–8]. As a natural component of human breast milk, nervonic acid is currently being used for infant formula supplementation [9–11]. As raw materials for industrial, pharmaceutical and nutraceutical applications, effective production of VLCFAs in oilseed, especially nervonic acid, has become an important goal of oilseed crop breeding [12–15].

Nervonic acid has been found only in the seed oils of a few known plants, primarily species of the Brassicaceae and several other family, including *Lunaria annua* (honesty), *Borago officinalis* (borage), *Cannabis sativa* (hemp), *Acer truncatum* (Purpleblow Maple), *Tropaeolum speciosum* (flame flower), *Cardamine graeca* (bittercress) and *Malania oleifera* [12–14, 16, 17]. Among these species, only *Lunaria annua* has been considered as a niche crop for future development. However, this plant is a biennial with highly variable seed yields (800–2,000 kg/ha) and the seed shattering problem. Hence, it is uneconomical to use *L*. *annua* for nervonic acid production yet [5], and it is highly demanded to improve the production of nervonic acid in other oilseed crops via metabolic engineering technology.

**Fig 1:**
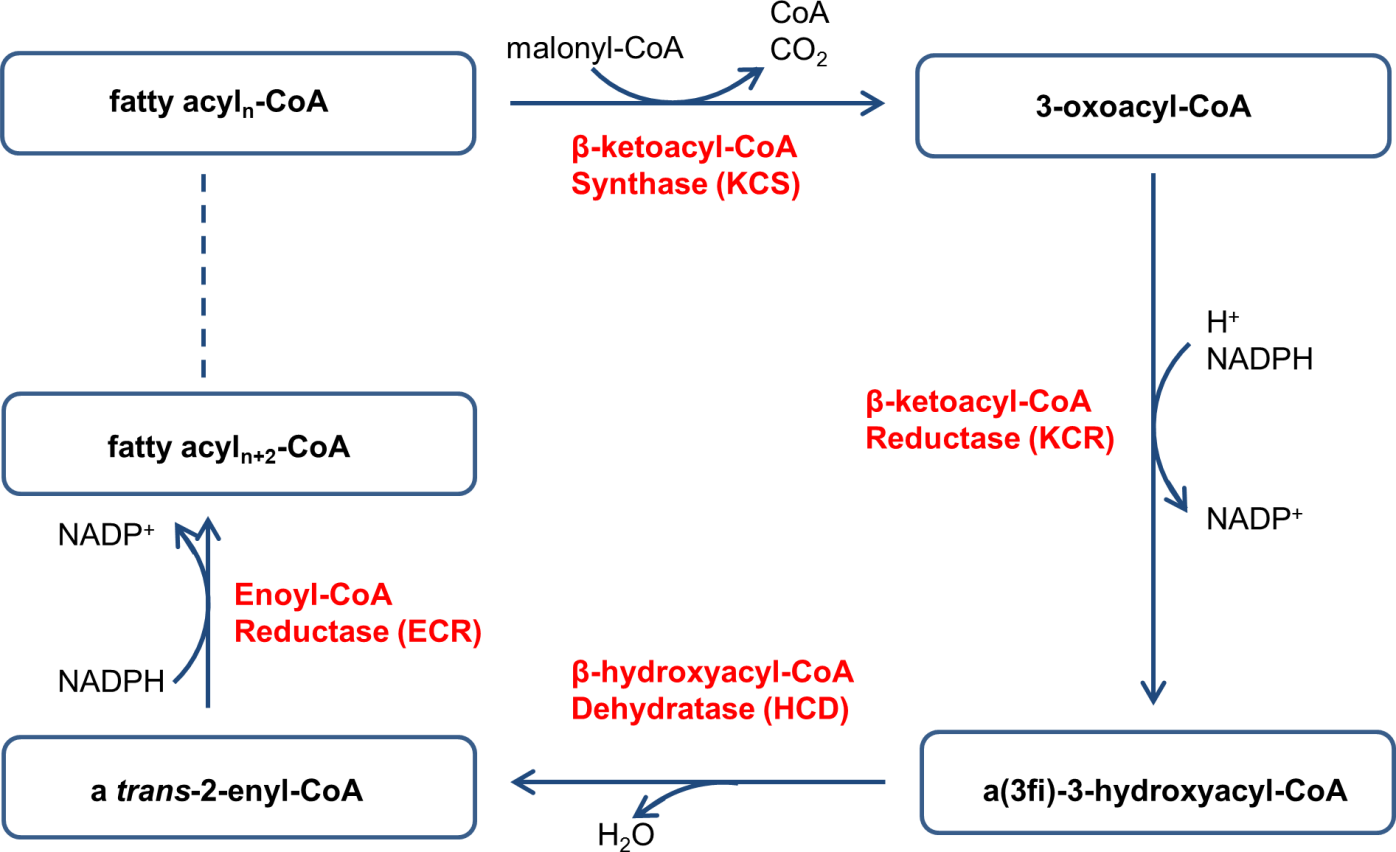
Elongation of Fatty Acids.

VLCFAs are synthesized in the endoplasmic reticulum (ER) by a membrane-bound fatty acid elongation complex that elongates C16 and C18 fatty acids, which are formed by the cytosolic fatty acid synthase complex. The elongase complex catalyzes the cyclic addition of two carbon units to the acyl chain, which involves four enzymatic reactions: condensation of long-chain acyl-CoA with malonyl-CoA by *β*-ketoacyl-CoA synthase (KCS) that results in a 3-ketoacyl-CoA, reduction of 3-ketoacyl-CoA to a 3-hydroxyacyl-CoA by *β*-ketoacyl-CoA reductase (KCR), dehydration of 3-hydroxyacyl-CoA to a 2-enoyl-CoA by *β*-hydroxyacyl-CoA dehydratase (HCD), and reduction of 2-enoyl-CoA by enoyl reductase (ECR) that generates an elongated acyl-CoA (Fig 1) [18–20]. KCS is considered to be the rate-limiting enzyme in fatty acid elongation, because it determines the substrate and tissue specificities of fatty acid elongation. Furthermore, regulating the expression of *KCS* affects the ultimate contents of VLCFAs [21–28]. In contrast, the other three enzymes are found to have broad substrate specificity and to be shared by all tissues exhibiting VLCFA biosynthesis [29–32]. *KCR*, *HCD* and *ECR* genes were successively identified and functionally characterized from *Arabidopsis thaliana* [30–32]. KCR1 (AtKCR) is the only *β*-ketoacyl-CoA reductase in *Arabidopsis*. Loss of KCR1 function leads to embryo lethality, while a partial loss of its function is associated with a general reduction of VLCFA contents in seed triacylglycerols, sphingolipids, cuticular waxes and root glycerolipids [30]. HCD, an essential protein for plants, is encoded by the *PASTICCINO2* (*AtHCD*) gene in *Arabidopsis*. Loss-of-transcript alleles of *pas2* are embryo lethal, and the knock-down mutants displayed a strong reduction but not complete absence of VLCFAs [31]. *ECERIFERUM10* (*AtECR*) is identified as a gene coding ECR in *Arabidopsis*. The absence of ECR activity results in a reduction of cuticular wax load and the VLCFA content of seed triacylglycerols and sphingolipids [32]. However, the effects of the other three enzymes in enhancing VLCFA content have not yet been investigated.

Camelina (*Camelina sativa*) is an emerging Brassicaceae oilseed crop with many favorable agronomic attributes, such as broad distribution, cold and drought tolerance, relatively low fertilizer requirement, short life span (100–120 days) and easy transformation using *Agrobacterium*-mediated floral dip method [33–36]. In light of these properties, camelina is gaining popularity as an industrial platform for engineering fatty acid oil production [37–42].

The present study was aimed to systematically assess the roles of the other three enzymes of ER fatty acid elongation complex in raising the content of VLCFAs in oilseeds. As it is absent in conventional camelina seed oil, nervonic acid was chosen for the evaluation of the combinatorial effects of fatty acid elongase enzymes. A *LaKCS* gene from *L*. *annua*, which has been identified to be responsible for nervonic acid synthesis [12], and other three genes (*AtKCR*, *AtHCD* and *AtECR*) from *Arabidopsis* were selected as candidate genes. A previously modified extensive metabolic engineering tool box of seed-specific promoters and selection markers was used to facilitate this study [37, 43]. Nervonic acid content in seeds was analyzed to evaluate the effects of five different combinations on VLCFA production: *LaKCS* alone, *LaKCS* with *AtKCR*, *LaKCS* with *AtHCD*, *LaKCS* with *AtECR*, *LaKCS* with *AtKCR* and *AtHCD*. This study also attempted to develop camelina lines with a high nervonic acid content for potential pharmaceutical and nutraceutical applications.

## Materials and Methods

### Plant materials and growth conditions

Camelina (*Camelina sativa* cv. Sunesson) plants were grown under greenhouse conditions with 14 h day length (24–26°C) and 8 h dark (18–20°C) with natural and supplemental lights at 400–500 μmoles/m^2^/s as previously described [37].

### Vector construction

**Fig 2:**
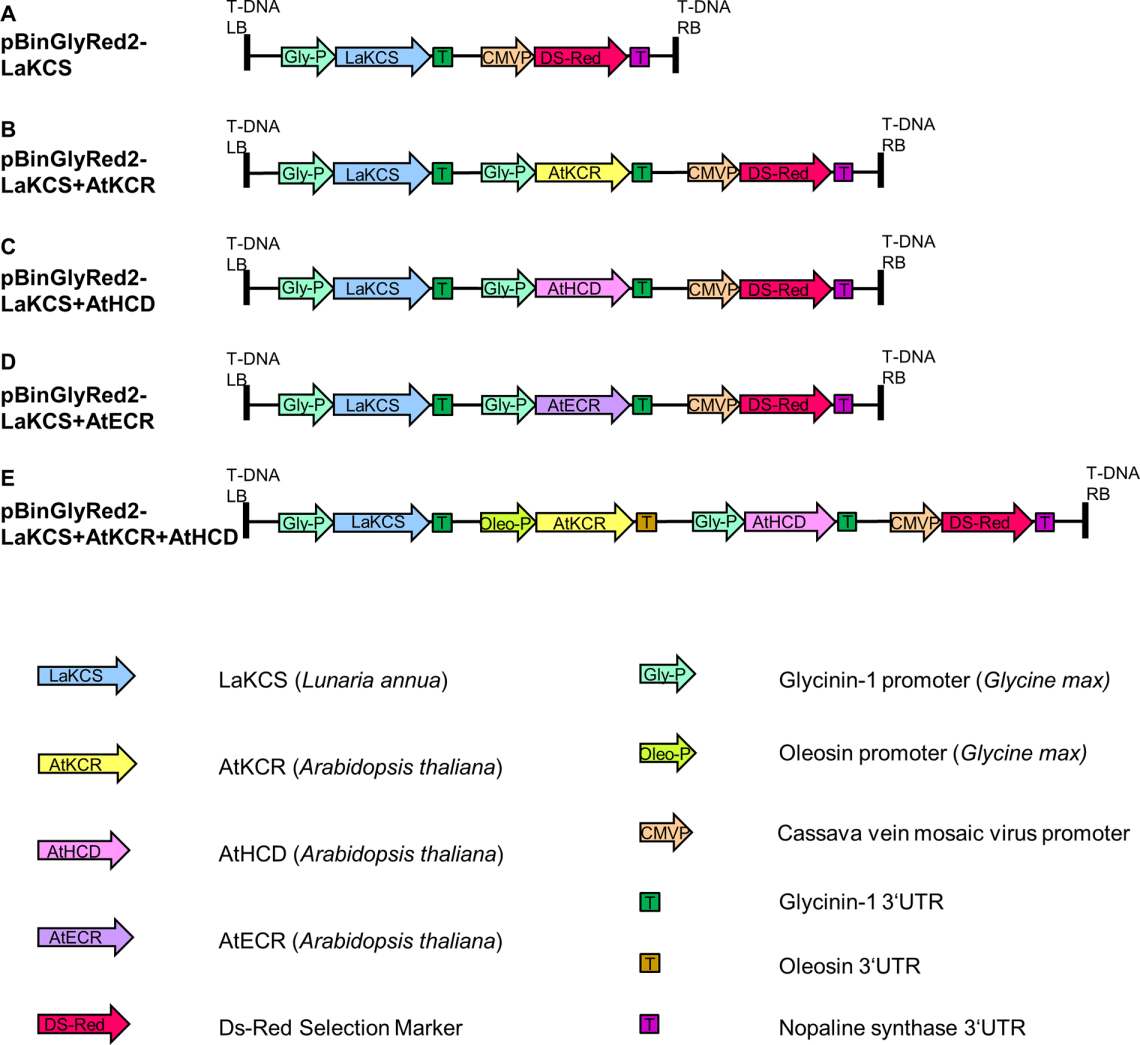
Transgenes used for generating nervonic acids in camelina seeds. (A). pBinGlyRed2-LaKCS contains seed-specific cassettes for the expression of *LaKCS* gene. (B). pBinGlyRed2-LaKCS+AtKCR contains seed-specific cassettes for the expression of *LaKCS* and *AtKCR* genes. (C). pBinGlyRed2-LaKCS+AtHCD contains seed-specific cassettes for the expression of *LaKCS* and *AtHCD* genes. (D). pBinGlyRed2-LaKCS+AtECR contains seed-specific cassettes for the expression of *LaKCS* and *AtECR* genes. (E). pBinGlyRed2-LaKCS+AtKCR+AtHCD contains seed-specific cassettes for the expression of *LaKCS*, *AtKCR* and *AtHCD* genes. Selection was accomplished with a DsRed florescence marker. Constitutive and seed-specific promoters, 3’UTR sequences and arrangements of cassettes are also shown.

Primers for KCS were designed based on *L*. *annua KCS* (EU871787). The *LaKCS* gene was amplified by PCR from *L*.*annua* developing seed cDNA using the following primers with added *Not*I restriction sites: 5’-CATGGCGGCCGCATGACGTCCATTAACGTAAAG-3’ and 5’-CATGGCGGCCGCTTAGGACCGACCGTTTTGGGC-3’ (the added restriction sites are underlined). The *Not*I digested fragment was ligated into the vector pKMS3 [38] to place *LaKCS* under the control of seed-specific soybean glycinin-1 promoter and 3’UTR to create pKMS3-LaKCS. A cassette comprising the glycine-1 promoter and 3’UTR flanking *LaKCS* gene was excised using *Asc*I and cloned into the binary vector pBinGlyRed2 containing a DsRed marker gene [38] to generate pBinGlyRed2-LaKCS (Fig 2A).

The *Arabidopsis* genes, *AtKCR* (At1g66730), *AtHCD* (At5g10480) and *AtECR* (At3g55360), were amplified from a cDNA library prepared from developing seeds using the following primers: 5’-CATGGAATTCATGGAGATCTGCACTTACTTC-3’ (*Eco*RI) with 5’-CATGCTCGAGTCATTCTTTCTTCATGGAGTC-3’ (*Xho*I) for *AtKCR* gene cloning; 5’-CATGGAATTCATGGCGGGCTTTCTCTCCGTT-3’ (*Eco*RI) with 5’-CATGGTCGACTTATTCCCTCTTGGATTTGGA-3’ (*Sal*I) for *AtHCD* gene cloning; and 5’-CATGGAATTCATGAAGGTCACCGTCGTCTCC-3’ (*Eco*RI) with 5’- CATGCTCGAGCTAAAGGAATGGAGGAAGTAT-3’ (*Xho*I) for *AtECR* gene cloning. The PCR products of *AtKCR* and *AtECR* were digested with *Eco*RI and *Xho*I, while the *AtHCD* PCR product was digested with *Eco*RI and *Sal*I. The digested fragment of each gene was linked into the corresponding site of pBinGlyRed2 vector, respectively. As a result, each gene was controlled by the seed-specific soybean glycinin-1 promoter and 3’UTR. Based on the inserted gene, the resulting plasmids were designated as pBinGlyRed2-AtKCR, pBinGlyRed2-AtHCD and pBinGlyRed2-AtECR. Then the previously described *Asc*I fragment containing the complete expression cassette of *LaKCS* was cloned into the corresponding sites of pBinGlyRed2-AtKCR, pBinGlyRed2-AtHCD and pBinGlyRed2-AtECR, respectively. The final vectors were designated as pBinGlyRed2-LaKCS+AtKCR (Fig 2B), pBinGlyRed2-LaKCS+AtHCD (Fig 2C) and pBinGlyRed2-LaKCS+AtECR (Fig 2D).

To generate the vector simultaneously expressing *KCS*, *KCR* and *HCD*, the *AtKCR* gene was amplified by PCR using the primers with added *Not*I restriction sites: 5’-CATGGCGGCCGCATGGAGATCTGCACTTACTTC-3’ and 5’-CATGGCGGCCGCTCATTCTTTCTTCATGGAGTC-3’. The *Not*I digested fragment was ligated into the vector pKMS2 with a strong seed-specific soybean oleosin promoter and 3’UTR and was named as pKMS2-AtKCR. A cassette harboring the oleosin promoter, *AtKCR* open reading frame and the oleosin 3’UTR was restricted by *Asc*I and cloned into *Mlu*I restriction sites of pBinGlyRed2-LaKCS+AtHCD to obtain the final vector pBinGlyRed2-LaKCS+AtKCR+AtHCD (Fig 2E).

### Camelina transformation and selection of transformants

Vectors of pBinGlyRed2-LaKCS, pBinGlyRed2-LaKCS+AtKCR, pBinGlyRed2-LaKCS+AtHCD, pBinGlyRed2-LaKCS+AtECR and pBinGlyRed2-LaKCS+AtKCR+AtHCD were transformed into *Camelina sativa* by the *Agrobacterium*-mediated floral vacuum infiltration method individually [35]. DsRed-positive seeds were identified using a green LED flashlight with a red camera filter lens [35].

### Gas chromatographic analysis of fatty acid compositions

Fatty acid methyl esters (FAMEs) were prepared from 25 mg DsRed positive mature camelina seeds with 2.5% (v/v) sulfuric acid/methanol as previously described [44]. For developing seeds, 20 seeds from an individual plant were collected to be analyzed as a sample, and 3–4 samples were prepared for each transgenic T_3_ line. FAMEs were analyzed using an Agilent 6890 gas chromatograph with flame ionization detection as previously described [44]. Fatty acids, including nervonic acid (C24:1), were identified by retention time according to previously studies [12, 13].

### Quantitative real-time PCR (qRT-PCR) analysis

Total RNA was extracted from immature seeds at 25 days after flowering (DAF) using a method described by Suzuki *et al*. [45]. First-strand cDNA was synthesized from 2 μg of total RNA using the Invitrogen ThermoScript RT-PCR system. Primers (S1 Table) were designed using the IDT DNA Real Time PCR primer design tool (http://www.idtdna.com/scitools/Applications/RealTimePCR). Real-time PCRs were performed using paired samples with three technical replicates on a Bio-Rad CFX96 Real-Time system (Bio-Rad, http://www.bio-rad.com) and DBI Bioscience Bestar-Real Time PCR Master Mix kit following the manufacturer’s instructions. The data were analyzed with LINREG as previously described [46]. The experiment was repeated using at least three independent biological replicates, with three technical replicates for each biological sample.

### Acyl-CoA profiling

Acyl-CoAs were extracted from pooled and lyophilized dry residues of the developing seeds 25 DAF from transgenic plants and wild-type plants. The samples were prepared and analyzed by electrospray ionization-mass spectrometry as described by Kim *et al*. [47].

### Statistical analysis

For pare-wise comparison, we used t-test. For the comparison of multiple means, the test for statistical significance was performed with ANOVA and Fisher’s least significant difference (LSD) multiple-comparison test using IBM SPSS Statistics software. In all the analyses, only P < 0.05 was considered statistically significant and different probability levels were calculated.

## Results

### Characterization of transgenic plants

To examine the effects of different combinations of fatty acid elongase enzymes, five constructs respectively harboring (i) only *LaKCS*, (ii) *LaKCS* and *AtKCR*, (iii) *LaKCS* and *AtHCD*, (iv) *LaKCS* and *AtECR*, and (v) *LaKCS*, *AtKCR* and *AtHCD* were used for transforming camelina plants. At least seven transgenic lines were obtained for each construct. All transgenic camelina plants were first selected by visible DsRed marker and verified by PCR for the respective transgenes. None of the transgenic lines exhibited any growth anomalies, and no apparent penalty on plant vigor or seed yield was observed under greenhouse and growth chamber conditions.

**Fig 3:**
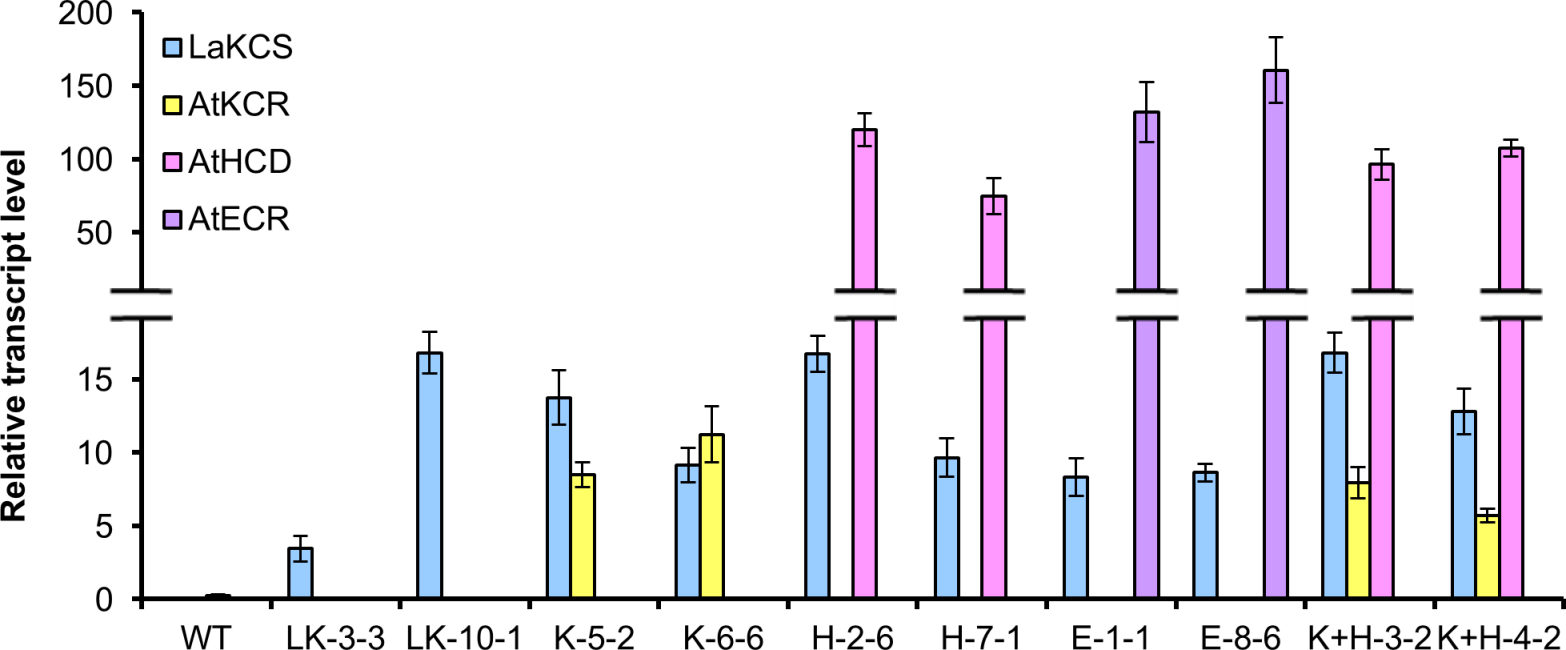
Relative transcript levels of transgenes in immature seeds from T_3_ engineered camelina lines and the non-transgenic control (Wt). LK-3-3 and LK-10-1 are the homozygous T_3_ lines transformed with only *LaKCS*. K-5-2 and K-6-6 are the homozygous T_3_ lines transformed with *LaKCS* and *AtKCR*. H-2-6 and H-7-1 are the homozygous T_3_ lines transformed with *LaKCS* and *AtHCD*. E-1-1 and E-8-6 are the homozygous T_3_ lines transformed with *LaKCS* and *AtECR*. K+H-3-2 and K+H-4-2 are the homozygous T_3_ lines transformed with *LaKCS*, *AtKCR* and *AtHCD*. The relative expression values of genes were measured on developing seeds harvested at 25 days after flowering. *CsACTIN7* gene expression level was used as a constitutive control. Values are the means and SE of three independent biological replicates.

qRT-PCR analyses on the developing seeds of the highest nervonic acid-accumulating transgenic T_3_ lines were performed to determine the transcripts of the four target transgenes. The expression levels of the four transgenes in the non-transformed control were extremely low and not detectable (Fig 3). All the four target genes were expressed as expected in the transgenic lines (Fig 3).

### Expression of *LaKCS* significantly increases nervonic acid content in camelina seeds

Eighteen *LaKCS*-expressing T_1_ lines were obtained and the fatty acid composition of the DsRed positive seeds from each line was determined. We observed significant differences in fatty acid composition in comparison with the non-transgenic control (S2 Table). Nervonic acid was not detected in the seeds from Wt, while significant accumulation of nervonic acid was found in seeds from *LaKCS*-expressing lines. Two *LaKCS*-expressing T_1_ lines (LK-3 and LK-10) with a higher nervonic acid content were selected for further evaluation (S2 Table). A stable enhancement of nervonic acid and erucic acid content, accompanied with a significant reduction in eicosenoic acid (C20:1Δ11), was observed from T_1_ to T_3_ generations (Table 1, S2 and S3 Tables). The maximum nervonic acid content in the seeds from LK-10-1 amounted up to 12.8% in T_3_ generation, with an average level of 12.0% (Table 1 and S4 Table). A 2- to 3-fold increase in a small proportion of the saturated VLCFAs (C22:0 and C24:0) at the expense of C20:0 was also observed (Table 1).

**Table 1:** Total fatty acid composition (wt% of total fatty acids) in the seeds of T_3_ camelina transgenic lines.

Line	Transformed genes	C16:0	C18:0	C18:1	C18:2	C18:3	C20:0	C20:1	C22:0	C22:1	C24:0	C24:1	VLCFA*
Control	None	7.2±0.6cd	3.2±0.7a	11.6±1.2a	21.2±2.3bc	34.9±3.2a	1.9±0.5a	13.1±05a	0.4±0.1d	3.4±0.3c	ND	ND	18.8±0.7d
LK-3-3	*LaKCS*	10.8±0.6a	3.0±0.4a	6.5±0.5cd	29.2±2.2a	25.2±2.2c	1.0±0.2b	3.4±0.2b	1.5±0.1ab	10.5±1.3b	2.0±0.1f	5.9±0.6d	24.2±1.5c
LK-10-1	*LaKCS*	8.5±0.3b	1.6±0.2b	5.3±0.3d	19.2±1.7bcd	34.4±1.8a	0.7±0.1b	2.2±0.1def	0.9±0.1c	11.6±0.3a	2.8±0.1bc	12.0±0.7ab	30.2±0.8ab
K-5-2	*LaKCS* and *AtKC*R	6.6±0.3de	1.6±0.2b	8.6±0.8b	17.9±2.0bcd	35.3±1.9a	0.6±0.2b	2.1±0.1ef	1.1±0.3bc	11.2±0.2a	2.6±0.4bcd	11.8±0.4ab	29.4±0.3ab
K-6-6	*LaKCS* and *AtKC*R	6.4±0.3e	1.6±0.2b	7.7±0.2bc	18.9±1.0bcd	35.5±1.2a	0.6±0.1b	2.4±0.5cdef	1.2±0.2bc	11.5±0.5a	2.1±0.1ef	11.3±1.3bc	29.0±0.8b
H-2-6	*LaKCS* and *AtHCD*	8.3±0.2b	2.3±0.2ab	8.2±0.7bc	20.3±0.7bcd	28.9±1.8bc	0.5±0.1b	1.8±0.3f	1.4±0.2abc	11.5±0.4a	3.3±0.3a	12.9±0.7a	31.5±1.3a
H-7-1	*LaKCS* and *AtHCD*	8.8±0.3b	2.7±0.4a	8.8±0.8b	21.6±0.9b	28.8±1.0bc	0.6±0.2b	2.2±0.3ef	1.1±0.4bc	11.2±0.8a	2.6±0.2bcde	10.7±0.7bc	28.3±1.0b
E-1-1	*LaKCS* and *AtECR*	7.4±0.3cd	2.9±0.6a	8.6±0.7b	19.0±1.5bcd	31.5±2.0ab	0.7±0.1b	3.0±0.4bc	1.1±0.2bc	11.9±0.6a	2.3±0.1cdef	10.0±0.8c	29.1±0.2b
E-8-6	*LaKCS* and *AtECR*	7.5±0.2c	2.7±0.3a	8.5±0.7b	17.1±2.5cd	33.8±3.5ab	0.6±0.1b	2.9±0.1bcd	1.3±0.1abc	12.5±0.2a	2.1±0.1def	9.9±0.1c	29.4±0.3ab
H+K-3-2	*LaKCS*, *AtKCR* and *AtHCD*	8.5±0.3b	1.7±0.5b	7.3±0.6bc	19.0±0.7bcd	33.2±1.0ab	0.7±0.1b	2.1±0.1ef	1.7±0.1a	12.1±0.3a	3.0±0.4ab	10.0±0.3c	29.6±0.8ab
H+K-4-2	*LaKCS*, *AtKCR* and *AtHCD*	7.4±0.1cd	2.5±0.1ab	8.6±0.3b	16.7±1.4d	34.9±1.3a	0.6±0.1b	2.6±0.3cde	1.5±0.1ab	11.4±0.4a	2.3±0.2cdef	10.8±0.3bc	29.2±0.6ab

ND, not detected.

*VLCFA = C20:0+C20:1+C22:0+C22:1+C24:0+C24:1.

The values shown are analyses of 30 seeds from 3 to 4 independent measurements.

Means±SD followed by different letters in each column are statistically different at P<0.05 based on ANOVA and Fisher’s least significant difference (LSD) multiple-comparison.

The content of C24:1 in seeds of LK-10-1 was set as the standard for evaluation of other gene combinations, even though we only got one line with nervonic acid content was over 10%. Because the transcription level of LaKCS in LK-10-1 was close to that of other transgenic lines, but the transcription level of LaKCS in LK-3-3 was much lower compared with that of other transgenic lines (Fig 3). In addition, if more transformed lines gained, more lines with nervonic acid content over 10% would be obtained.

### Co-expression of *LaKCS* with one of the other three elongase is unable to further increase the nervonic acid content in camelina seeds

To examine the combinatorial effects of LaKCS with the other three enzymes separately, one of the three elongases, i.e. *AtKCR*, *AtHCD* and *AtECR*, were seed-specifically co-expressed with *LaKCS* gene in camelina, respectively. The fatty acid composition of the DsRed positive seeds from each line was determined.

Nine T_1_ lines co-expressing *LaKCS* and *AtKCR* were acquired. Subsequently, two lines (K-5 and K-6) with higher levels of nervonic acid were analyzed (S2 Table). Stable increases of nervonic acid, erucic acid and VLCFA contents were observed in three successive generations (Table 1, S2 and S3 Tables). K-5-2, a T_3_ line, showed the highest content of nervonic acid of 12.2% (with an average level of 11.8%), which was not higher than that in LK-10-1 (Table 1 and S4 Table). The same phenomenon was observed in K-6-6 (Table 1). The contents of other VLCFAs in T_3_ lines (K-5-2 and K-6-6), such as C22:1, C22:0 and C24:0, also did not show further increase compared with LK-10-1 (Table 1). All these results suggest that *AtKCR* expression in combination with *LaKCS* does not further enhance the production of nervonic acid and other VLCFAs.

Seven individual transgenic lines harboring *LaKCS* and *AtHCD* genes were identified with DsRed seeds. Similarly, two T_1_ transgenic lines H-2 and H-7 with higher nervonic acid content were chosen for further study (S2 Table). Increases in nervonic acid, erucic acid and VLCFAs in the subsequent generations were observed (Table 1, S2 and S3 Tables). For example, the average nervonic acid content was 12.9% in T_3_ generation line H-2-6 with a maximum level up to 13.8% (Table 1 and S4 Table). Compared with that of LK-10-1, the nervonic acid contents of H-2-6 and H-7-1 were not significantly higher (Table 1). Content of other VLCFAs including erucic acid and saturated VLCFAs in H-2-6 and H-7-1 were increased to the level of LK-10-1(Table 1). No significant difference in the content of nervonic acid and other VLCFAs was detected among H-2-6, H-7-1 and LK-10-1, suggesting that *AtHCD* expression also does not promote the production of nervonic acid and other VLCFAs above levels achieved with *LaKCS* expression alone.

Eight T_1_ transgenic lines harboring *LaKCS* and *AtECR* genes were collected. Two lines (E-1 and E-8) with higher nervonic acid content were selected for further analysis (S2 Table). Increases of nervonic acid, erucic acid and VLCFAs in the succeeding generations were observed (Table 1, S2 and S3 Tables). In T_3_ generation, the maximum nervonic acid content in the seed oil from line E-1-1 was 10.8% (with an average level of 10.0%). The maximum and average level of nervonic acid content in E-1-1 and E-8-6 were relatively lower than those in LK-10-1 (Table 1 and S4 Table). The contents of other VLCFAs in E-1-1 and E-8-6 were also approximately equal to those in LK-10-1 (Table 1). No significant difference in the contents of nervonic acid and other VLCFAs was observed among E-1-1, E-8-6 and LK-10-1, indicating that *AtECR* expression has no effect in promoting the production of nervonic acid and other VLCFAs above levels achieved with *LaKCS* expression alone.

Based on the above results, it can be concluded that two-gene combinations (*LaKCS* with anyone of the other three genes) cannot enhance the nervonic acid synthesis, as none of the co-expressing lines of two genes showed elevated nervonic acid content in seed oil compared with the elite *LaKCS*-expressing line LK-10-1.

### Co-expression of *LaKCS*, *AtKCR* and *AtHCD* is still unable to further increase the nervonic acid content in camelina seeds

Subsequently, we investigated the effect of a three-gene combination on nervonic acid synthesis. Based on the previous studies on two-gene combinatorial effect, *AtKCR* and *AtHCD* were selected for this investigation in addition to *LaKCS*, as the combination of *LaKCS* with *AtKCR* or with *AtHCD* resulted in a relatively higher content of nervonic acid than the combination of *LaKCS* with *AtECR* (Table 1). The construct harboring *LaKCS*, *AtKCR* and *AtHCD* genes was transformed into wild-type camelina. Eight T_1_ transgenic lines were gained, and two of them (K+H-3 and K+H-4) with higher nervonic acid contents were selected for further evaluation (S2 Table). The contents of nervonic acid, erucic acid and VLCFAs were steadily increased in the next two generations (Table 1, S2 and S3 Tables). In T_3_ generation, the maximum nervonic acid content was determined as 11.1% in the elite line H+K-4-2 (with an average level of 10.8%) (Table 1 and S4 Table). There was no significant difference in nervonic acid content among H+K-3-2, H+K-4-2 and LK-10-1 (Table 1). In addition, the contents of erucic acid and saturated VLCFAs in K+H-3-2 and K+H-4-2 were also enhanced to the level of LK-10-1 (Table 1). These results demonstrate that the combination of *LaKCS* with *AtKCR* and *AtHCD* together also fails to promote the nervonic acid production.

It is unnecessary to examine the effects of combination of LaKCS with AtKCR and AtECR, combination of LaKCS with AtHCD and AtECR, and combination of all the four genes, because the nervonic acid content in the co-expressing lines of *LaKCS* and *AtECR* was relatively lower compared with that of the elite transgenic lines of other two two-gene combinations, and the combination of LaKCS with AtKCR and AtHCD together still failed to further increase the nervonic acid production.

### Acyl-CoAs profile of transgenic lines

**Fig 4:**
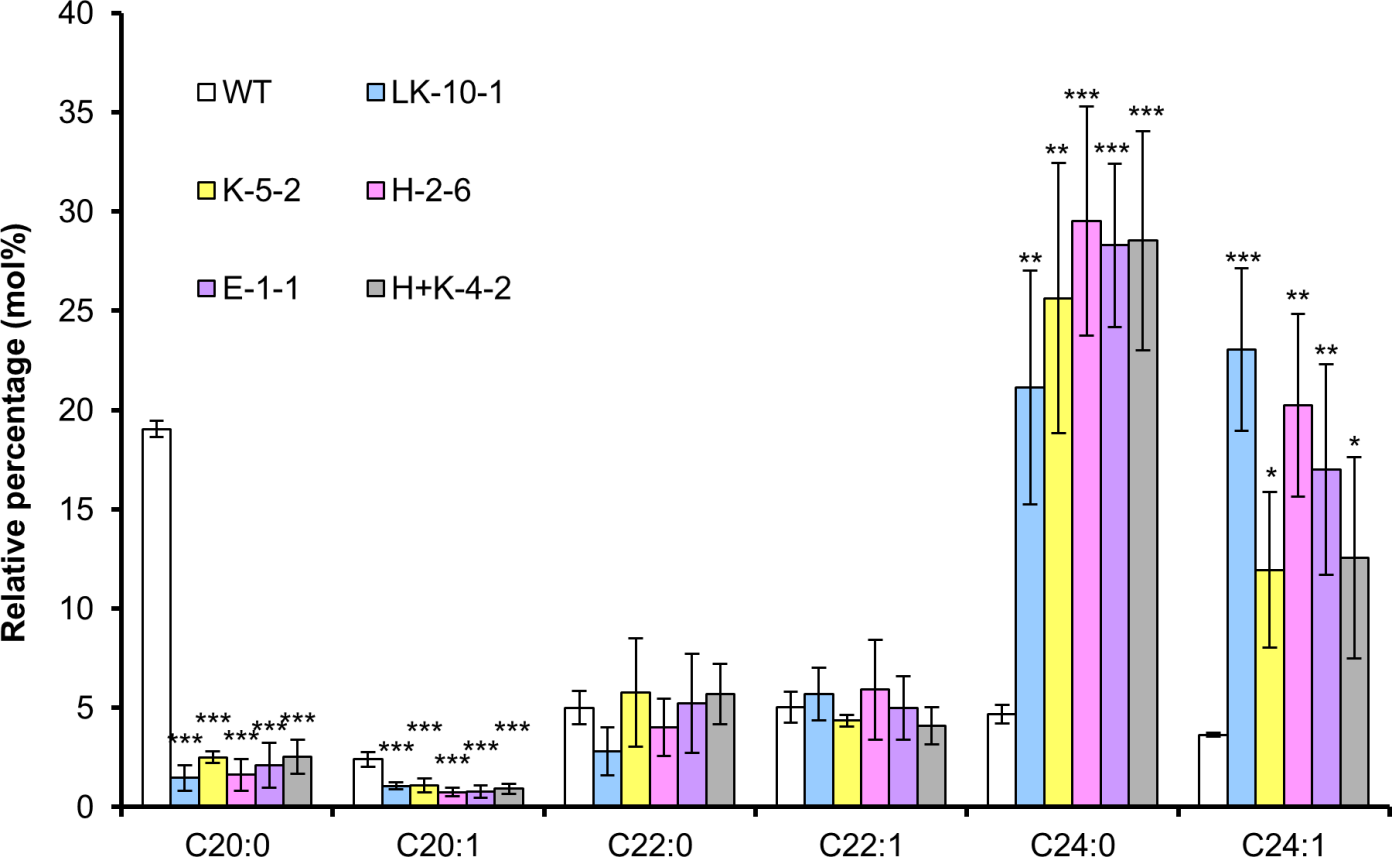
Fatty acyl-CoA profiling of non-transgenic control (Wt) and T_3_ engineered camelina lines. LK-10-1 is the homozygous T_3_ line transformed with *LaKCS*. K-5-2 is the homozygous T_3_ line transformed with *LaKCS* and *AtKCR*. H-2-6 is the homozygous T_3_ line transformed with *LaKCS* and *AtHCD*. E-1-1 is the homozygous T_3_ line transformed with *LaKCS* and *AtECR*. K+H-4-2 is the homozygous T_3_ line transformed with *LaKCS*, *AtKCR* and *AtHCD*. Data are presented as means with standard error bars SD, which were calculated based on 3–5 independent replicates. Samples from each replicate were measured using 15 mg developing seeds harvested at 25 days after flowering (DAF). Statistical significance for the difference between Wt and each transgenic line was determined by t-test. *, ** and *** designates a significant level at P < 0.05, P < 0.01 and P < 0.001, respectively.

The profile of fatty acyl-CoAs was analyzed from the developing seeds of transgenic T_3_ lines LK-10-1, K-5-2, H-2-6, E-1-1, H+K-4-2 and non-transformed control lines, as the nervonic acid contents in these transgenic lines were the highest. The contents of C22:0- and C22:1-CoAs were decreased, while the levels of C24:0- and C24:1-CoAs were substantially increased in all the transgenic T_3_ lines relative to the control (Fig 4). However, the C22:0- and C22:1-CoA contents were not significantly altered in all the transgenic T_3_ lines compared with in the control lines (Fig 4). These results confirm that the expression of *LaKCS* changed the contents of VLCFA-CoAs, while the VLCFA-CoAs composition would not be further changed by the co-expression of *LaKCS* with any one or two of the other fatty acid elongase genes.

### Fatty acid composition in developing seeds of transgenic lines

**Fig 5:**
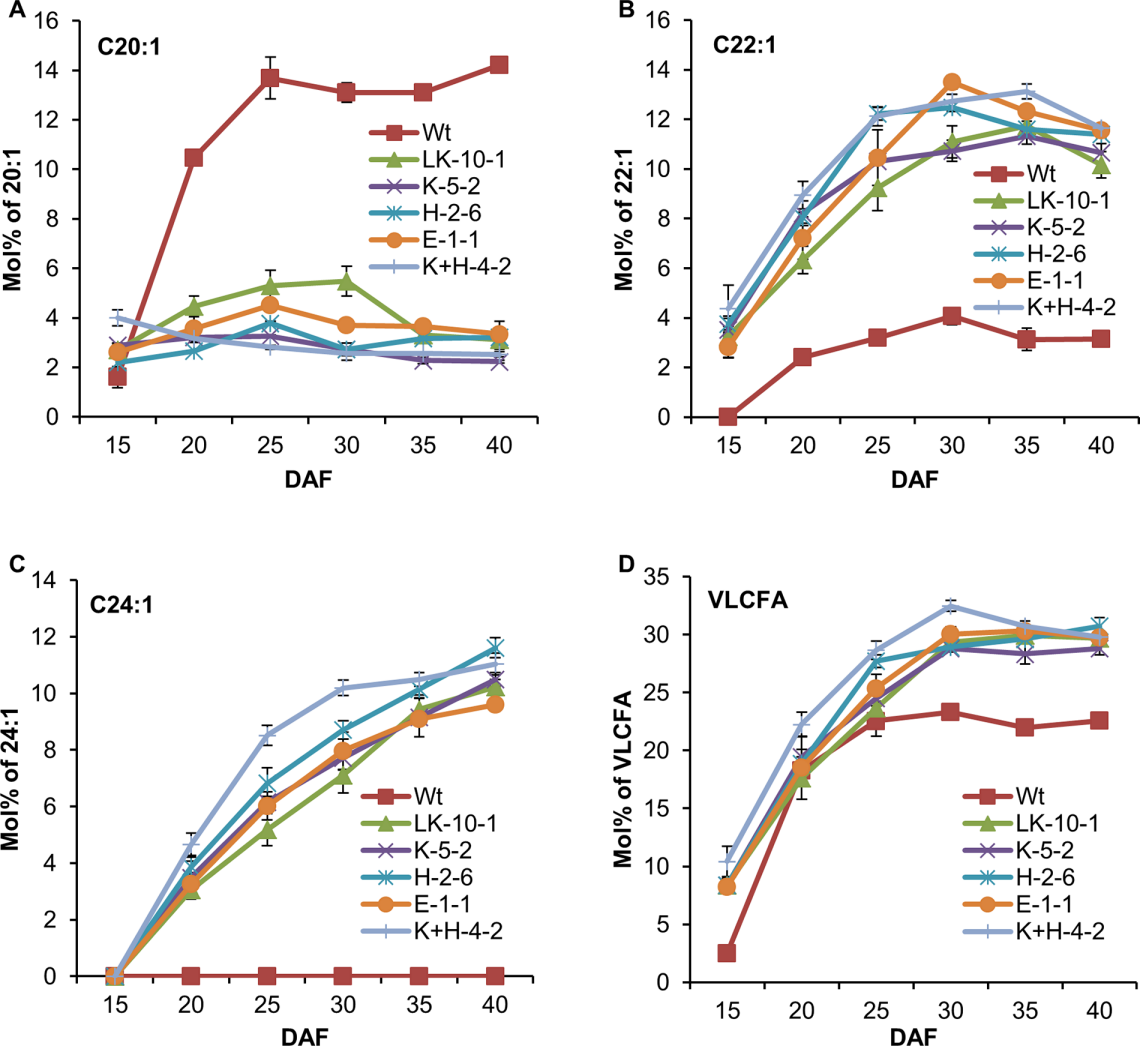
Fatty acid composition of seeds from non-transformed control (Wt) and T_3_ engineered camelina lines at different days after flowering (DAF). (A). C20:1 content; (B). C22:1 content; (C). C24:1 content; (D). VLCFA content. LK-10-1 is the homozygous T_3_ line transformed with only *LaKCS*. K-5-2 is the homozygous T_3_ line transformed with *LaKCS* and *AtKCR*. H-2-6 is the homozygous T_3_ line transformed with *LaKCS* and *AtHCD*. E-1-1 is the homozygous T_3_ line transformed with *LaKCS* and *AtECR*. K+H-4-2 is the homozygous T_3_ line transformed with *LaKCS*, *AtKCR* and *AtHCD*. Data are presented as means with standard error bars SD, which were calculated based on 3–4 independent replicates. Samples from each replicate were measured using 20 developing seeds harvested at 25 days after flowering (DAF). Statistical significance was evaluated by ANOVA and Fisher’s least significant difference (LSD) multiple-comparison. The complete analyses are presented in Supporting Information S5 Table.

Analysis of fatty acid composition in the developing seeds of non-transformed control (Wt) and transgenic T_3_ lines was conducted at 15, 20, 25, 30, 35 and 40 DAF. LK-10-1, K-5-2, H-2-6, E-1-1 and H+K-4-2 were selected to represent each combination because they displayed the highest nervonic acid content. The complete analyses are presented in Supporting Information S5 Table. Some of the data are extracted and presented in graphic form in Fig 5.

The content of C20:1 of control lines was sharply increased during 15–25 DAF, and then was gradually decreased during 25–35 DAF followed by a slight growth in the last 5 days (Fig 5A). However, the content of C20:1 in LK-10-1, K-5-2, H-2-6 and E-1-1 moderately increased during 15–25 DAF, and then gradually decreased during the rest period except for LK-10-1, whose C20:1 content reached the peak at 30 DAF (Fig 5A). Furthermore, the content of C20:1 in H+K-4-2 showed a continuous reduction during the whole detection (Fig 5A). In addition, the contents of C20:1 in all the transgenic lines were around 2–5%, whereas that of the control was as high as 13–14% (S5 Table), indicating that the accumulation of C20:1 in transgenic lines was inhibited due to the continuous elongation of carbon chains by the expression of *LaKCS*.

The accumulation of C22:1 in all transgenic lines showed the same trend as in the control. During 15–30 DAF, the content of C22:1 dramatically rose to a peak (10–13% in transgenic lines and 4% in control lines), and then slightly decreased during 30–40 DAF (Fig 5B; S5 Table).

The fatty acid C24:1 was not detected during seed development in the control (Fig 5C). The content of C24:1 continuously increased to 9–11% at 40 DAF in all transgenic lines (S5 Table). The C24:1 accumulation rates in K-5-2, H-2-6 and E-1-1 were slightly higher than that in LK-10-1 at some sampling points (Fig 5C; S5 Table). The C24:1 accumulation rate in H+K-4-2 was the highest at all sampling points, particularly during the earlier stages (20–30DAF) (Fig 5C; S5 Table), suggesting that simultaneous expression of *LaKCS* with *AtKCR* and *AtHCD* may significantly accelerated the accumulation of C24:1 at earlier stages of seed development.

The VLCFA content in the control increased during 15–25 DAF, and then plateaued at 22–23% (Fig 5D; S5 Table). Similar patterns were observed in the VLCFA accumulations of all transgenic lines (Fig 5D). The ultimate VLCFA contents of all transgenic lines were between 28% and 30% (S5 Table).

Taken together, the above results showed that the combination of *LaKCS* with KCR and HCD may accelerate the accumulation of nervonic acid during the seed development, but does not alter the final levels of nervonic acid accumulation.

## Discussion

VLCFA biosynthesis is controlled by a multienzyme complex known as fatty acid elongase, which is composed of KCS, KCR, HCD and ECR. Previously, only KCS was over-expressed to increase the VLCFA content, which showed that KCS is a rate-limiting enzyme for its substrate and tissue specificity [23, 28, 29, 48, 49]. However, the impact of the other three genes on VLCFA production is still not clear from these past reports. In this study, we systematically evaluated the combinatorial effects of KCR, HCD and ECR with KCS on VLCFA production.

The *LaKCS* gene from *L*. *annua* has been identified to be responsible for nervonic acid synthesis [12], but the other three component of the elongase complex gene, *KCR*, *HCD* and *ECR* in *L*. *annua* have not been identified yet. In contrast, *AtKCR*, *AtHCD* and *AtECR* in *Arabidopsis*, only have one functional copy for each enzyme and have broad substrate specificity, and different KCSs shared the other three components to form elongase [30–32]. Taken together, we chose to use *AtKCR*, *AtHCD* and *AtECR* with *LaKCS* to study the combinatorial effects of all the elongase components.

Compared with that in *LaKCS*-expressing lines, the nervonic acid content in two-gene co-expressing lines was not further increased (Table 1), indicating that the combinations of *LaKCS* with another elongase gene cannot promote the nervonic acid production. Then *AtKCR* and *AtHCD* were co-expressed with *LaKCS* in camelina seeds, but the nervonic acid content still was not enhanced (Table 1). The combination of *LaKCS* with *AtKCR* and *AtHCD* also failed to promote the nervonic acid production. We did not examine the effects of combination of *LaKCS* with *AtKCR* and *AtECR*, combination of *LaKCS* with *AtHCD* and *AtECR*, and combination of all the four genes, because the nervonic acid content in the top nervonic acid accumulating-co-expression lines of *LaKCS* and *AtECR* was relatively lower compared with that of the top nervonic acid accumulating- lines of other two two-gene combinations.

This study further confirms that KCS is the rate-limiting enzyme in the VLCFA synthesis. KCS catalyzes the first step of elongation, and the products of KCS are the precursors of KCR, HCD and ECR. As a result, the amount of ultimate product is determined by the amount of KCS, and is not affected by the amount of KCR, HCD and ECR.

Interestingly, nervonic acid accumulation in the seeds from the transgenic line of co-expressing *LaKCS* with *AtKCR* and *AtHCD* appeared to occur more rapidly during seed development than that in the seeds from *LaKCS*-expressing lines (Fig 5C). It is likely that overexpression of KCS together with KCR and HCD might result in more efficient successive catalyzes of the intermediate products. However, such a difference in mature seeds is diminished and the final content of nervonic acid in all transgenic plants did not show significant differences. Further studies are required to understand why such a developmental change could happen. In this regard, identification of all the endogenous *CsKCS*, *CsKCR*, *CsHCD* and *CsECR* genes in camelina shall be the first step for further analysis of the interaction among the transgenes and endogenous genes.

It was noticed that there was a more than ten-fold difference in the transcription levels of the *AtKCR* gene in K-5-2 and *AtHCD* in H-2-6 that are under the control of the same Glycinin-1 promoter (Fig 3). In addition, the *LaKCS* gene and *AtHCD* genes in transgenic lines of K+H-3-2 and K+H-4-2, which were transformed with the same construct, are separately driven by Glycinin-1 promoter, but the transcription level of *AtHCD* was much higher than that of *LaKCS* (Fig 3). On the other hand, the same gene under the control of different promoters may exert similar transcription level. For example, the *AtKCR* gene is controlled by Glycinin-1 promoter in K-5-2 and by oleosin promoter in H+K-3-2, but the transcription level of them were almost the same (Fig 3). Such differences may be attributed to the properties of the transgenes themselves. Based on the expression data in *Arabidopsis* e-FP browser [50], the transcription levels of *AtHCD* and *AtECR* in *Arabidopsis* all are approximately 20-fold higher than that of *AtKCR* during seed development, indicated that *AtHCD* and *AtECR* are highly expressing genes while *AtKCR* is not. Such a property may be related to the mRNA stability and decay rate of individual mRNA molecules, which are influenced by GC% of gene sequence, the sequence elements of DNA or the secondary structure of mRNAs [51–53]. When a highly expressing gene was overexpressed in camelina seeds, they may keep the property to show higher transcription level. In addition, the stability and decay rate of mRNAs may affect the cDNA abundance of different genes [54–55]. As a result, different genes displayed different transcription levels even driven by the same promoter. In camelina, the overall content of nervonic acid was 10–13% in the seeds from the series of transgenic lines with *LaKCS* (Table 1). Heterologous expression of *LaKCS* gene in *Arabidopsis* and *Brassica carinata* increased the nervonic acid content to 3–5% and 20–30%, respectively [12]. The nervonic acid contents are comparatively lower in seed oil from engineered camelina and *Arabidopsis*. The content of erucic acid, which is the major precursor of nervonic acid, is low in the seeds from camelina and *Arabidopsis*. In contrast, in *B*. *carinata*, erucic acid substrate is present at higher levels for elongation. The above facts suggest that substrate availability and magnitude can influence the efficiency of chain elongation and eventually the contents of VLCFAs. Similar phenomenon was also observed in other studies, in which another *KCS* gene from *Cardamine graece* was ectopically expressed in *Arabidopsis*, *Brassica carinata* and *Brassica napus* [13].

The desired nervonic acid oils for nutraceutical, pharmaceutical and industrial applications are the one with a high content of nervonic acid but a very low content of erucic acid. The content of erucic acid in the oil from the camelina transgenic lines in this study was 10–12%, while the ideal oil should contain no more than 5% of erucic acid and should have a higher content of nervonic acid [13]. Thus, a more suitable balance between the contents of nervonic and erucic acid for commercial applications in camelina awaits further study.

## Supporting Information

S1 Table Primers used in cloning and real-time RT-PCR experiments. (XLSX)

S2 Table Total fatty acid composition of seeds from non-transformed and T_1_ elite engineered lines of camelina. (XLSX)

S3 Table Total fatty acid composition of seeds from non-transformed and T_2_ engineered lines of camelina. (XLSX)

S4 Table Total fatty acid composition of seeds from non-transformed and T_3_ engineered lines of camelina. (XLSX)

S5 Table Comparisons for C20:1, C22:1, C24:1 and VLCFA of non-transformed control (Wt) and T_3_ engineered camelina lines with the highest nevonic acid content at different days after flowering (DAF). (XLSX)
